# Phytochemicals, Bioactive Properties and Commercial Potential of Calamondin (*Citrofortunella microcarpa*) Fruits: A Review

**DOI:** 10.3390/molecules28083401

**Published:** 2023-04-12

**Authors:** Karthikeyan Venkatachalam, Narin Charoenphun, Pao Srean, Vasin Yuvanatemiya, Chinnawut Pipatpanukul, Kanokporn Pakeechai, Thanya Parametthanuwat, Jittimon Wongsa

**Affiliations:** 1 Faculty of Innovative Agriculture and Fishery Establishment Project, Prince of Songkla University, Surat Thani Campus, Makham Tia, Mueang 84000, Surat Thani, Thailand; karthikeyan.v@psu.ac.th; 2 Faculty of Science and Arts, Burapha University Chanthaburi Campus, Thamai 22170, Chanthaburi, Thailand; 3 Faculty of Agriculture and Food Processing, National University of Battambang, Battambang 020101, Cambodia; pao.srean@gmail.com; 4 Faculty of Marine Technology, Burapha University Chanthaburi Campus, Thamai 22170, Chanthaburi, Thailand; vasin@buu.ac.th; 5 Faculty of Engineering, Burapha University, Muang 20131, Chonburi, Thailand; chinnawut.pi@eng.buu.ac.th; 6 Faculty of Business Administration and Information Technology, Rajamangala University of Technology Suvarnabhumi, Phranakhon Si Ayutthaya 13000, Phranakhon Si Ayutthaya, Thailand; kanokporn.p@rmutsb.ac.th; 7 Department of Agricultural Engineering for Industry, Faculty of Industrial Technology and Management, King Mongkut’s University of Technology North Bangkok (Prachinburi Campus), Muang 25230, Prachinburi, Thailand; thanya.p@itm.kmutnb.ac.th (T.P.); jittimon.w@itm.kmutnb.ac.th (J.W.); 8 KMUTNB Techno Park Prachinburi, King Mongkut’s University of Technology North Bangkok (Prachinburi Campus), Muang 25230, Prachinburi, Thailand; 9 Food and Agro-Industry Research Center, King Mongkut’s University of Technology North Bangkok, Bangsue, Bangkok 10800, Thailand

**Keywords:** calamondin fruit, peel, phytochemicals, D-limomene, extraction, biological activities, byproducts

## Abstract

The calamondin (*Citrofortunella microcarpa)* is a hybrid citrus fruit resulting from the crossing of a mandarin orange with a kumquat. It is a small, round-shaped fruit with thin, smooth skin ranging from orange to dark red. The aroma of the fruit is distinctive and unique. Calamondin is an excellent source of Vitamin C, D-Limonene, and essential oils, providing benefits to the immune system, as well as anti-inflammatory, anti-cancer, anti-diabetic, anti-angiogenic, and anti-cancer properties, and it exhibits various therapeutic effects. It also contains a good amount of dietary fiber from pectin. Its distinctive flavor and high juice content make calamondin juice a popular ingredient in many international cuisines. The juice also contains bioactive compounds, such as phenolics and flavonoids, which are a potential source of antioxidant properties. All parts of the calamondin fruit, including the juice, pulp, seeds, and peel, can be used in various applications, from food products like juices, powders, and candies to non-food uses in herbal medicine and cosmetics, showcasing their versatility and unique properties. This review will examine various bioactive components of calamondin and their related medicinal effects, and provide guidelines for their utilization, processing, and value addition on a commercial scale.

## 1. Introduction

Calamondin (*Citrofortunella microcarpa)*, also known as Calamansi orange, is a hybrid citrus fruit cross between a kumquat and other citrus species. It is classified in the family Rutaceae, which includes plants such as lime, pomelo, and tangerine [1]. It is believed that calamondin originated in China and was then introduced to various regions, including Southeast Asia, India, Hawaii, the West Indies, Central America, and North America. Additionally, calamondin is often planted in the Philippines as an alternative to lime due to its tolerance of pests and diseases [2]. As a result of its resistance to pests and diseases, calamondin has become a crucial economic fruit in those countries, both for domestic consumption and as an export product [1]. The countries that import the most calamondin from Southeast Asia are the United States, Japan, South Korea, Canada, and Hong Kong. Calamondin fruit is commonly utilized in various ways in countries like Malaysia, Singapore, Vietnam, China, and Indonesia, for example, as an ingredient in cooking, medication, and medical cosmetics [1,2,3]. Furthermore, it can also be found in Europe and America, where it is being introduced as potted plants that decorate gardens and have a high commercial value. In many countries, calamondin fruits are mainly grown for household consumption and are sold when there is a surplus [3]. They are used as a substitute for lime in various dishes, such as shrimp paste sauce, salt, chili paste, pickled calamondin fruit, and calamondin fruit juice. Additionally, calamondin fruit juice is an inexpensive vitamin C source. Calamondin fruit comprises two parts: juice and juice byproducts (peel, seeds, pulp). The primary acids present in calamondin are ascorbic, dehydroascorbic, and citric acids. The peel and dregs of the fruit are rich in dietary fiber [1]. The peel also contains flavonoids, such as naringosi, hesperidin, diosmin, diosmetin, and hesperidin. The essential oils of calamondin fruit peel are clear liquids with a distinct fragrance. D-Limonene is the main constituent of the essential oils. The peel of calamondin has been found to have numerous biological and pharmacological benefits, including antioxidant, antibacterial, anti-inflammatory, anti-hyperglycemic, anti-diabetic, anti-angiogenic, and anti-tumor effects [1,2,3,4]. Acquiring knowledge about the various uses and value-added products of calamondin fruits is crucial for its commercialization and addressing malnutrition. This includes understanding its benefits, the chemical compounds it contains, and the potential value-added products that can be made from it to utilize it effectively in the future.

## 2. General Characteristics of Calamondin Fruit

**Figure 1:**
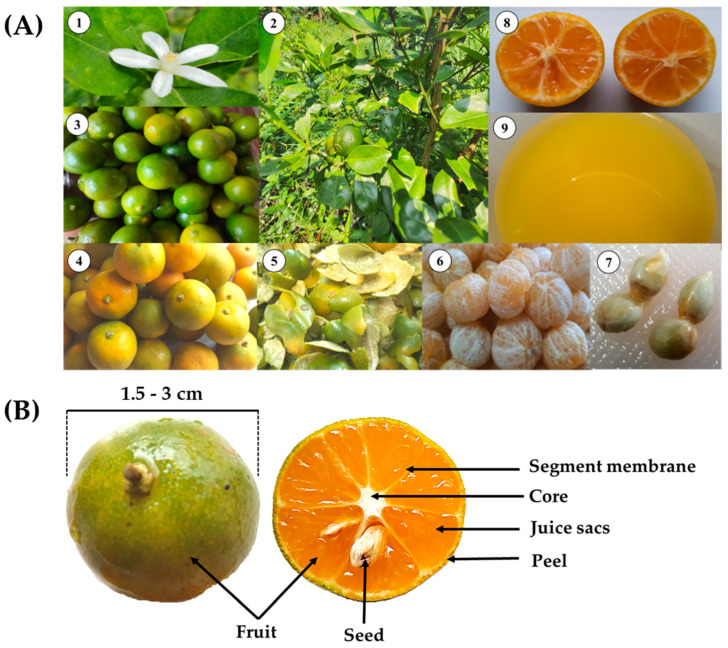
(**A**) General characteristics of calamondin: (**1**) flower, (**2**) stems and leaves, (**3**) unripe fruits, (**4**) ripe fruits, (**5**) peels, (**6**) peeled fruits, (**7**) seeds, (**8**) pulp, and (**9**) juice. (**B**) Calamondin fruit structure.

Calamondin is a tropical plant that is widely distributed and easy to grow in many tropical countries. The photographic illustration in Figure 1 shows the essential characteristics and structure of calamondin fruit. Calamondin can be propagated through sexual (seeds) and asexual (cutting, layering, budding, grafting) methods. It has a medium-sized shrub shape, reaching heights of 1.5–3 m, and its branches form dense bushes, giving it an attractive tree shape. Calamondin has oval-shaped leaves that measure 2–4 cm wide and 4–7 cm long, with a glossy surface. It flowers throughout the year, with single flowers appearing in short bouquets along the auxiliaries and branch ends, which are white and have a strong fragrance. Because of these features, it is often planted as an ornamental plant or a potted ornamental plant and bears fruits gradually throughout the year. Calamondin is similar to a lime because it produces more fruits during the rainy season than in the dry season. The fruit is round with a diameter of 1.5–3 cm, has a thin green skin that is fragrant, is divided into 7–10 segments, and has 1–7 seeds per fruit. As it ripens, the fruit turns a bright-orange color with a light-green tinge and is filled with juice [3].

Citrus trees typically have 4–5 growth flushes per year, each with the possibility of producing flowers and fruit. However, most citrus only flowers and sets fruit during the spring flush. Lemons, limes, kumquats, and calamondins are exceptions, as they can flower and produce fruit year round. Calamondin, often grown as a bonsai, constantly blooms, releasing a citrus blossom scent. It may have both fruit and flowers at the same time. Non-spring flush fruit, such as oranges, tangelos, and grapefruits, have a puffy appearance, thick skin, and sheep-nosed shape. Calamondin fruit matures slowly, enhancing its ornamental value in the landscape for longer than other citrus fruits. It can be harvested all year, with peak abundance from November–June and increased sweetness towards the end of the season. However, the thin skin makes calamondins perishable after harvesting. Calamondin fruit may take up to a year to ripen and turn orange. However, an orange color may indicate over-ripeness and loss of flavor. Smaller than a regular lime, with a thin skin, it is best consumed within a week of harvest if not refrigerated. It is recommended to use clippers or scissors to pick the fruit to prevent stem-end tearing, which hastens degradation [4].

The growth stages of calamondin’s flowers, fruit, and seeds, from initiation to commercial maturity, are described by time intervals. Rosillo-Magno and Mapalo [5] reported the morphological study of calamansi fruit, which is essential for crop-breeding programs. The floral, fruit, and seed sections were stained with 1% safranin solution, then examined under a dissecting microscope. Based on distinct morphological events, the development phases are divided into nine stages for flowers, three for fruit, and four for seeds. Flowers take 17 to 23 days from bud creation to anthesis. Fruit requires 78 to 84 days to mature from anthesis to commercial maturity. From flower bud creation to commercial maturity, fruit takes 94 to 101 days, and its skin becomes lustrous and smooth. Cross-pollination can be completed between 17 and 23 days after flower bud development. Immature fruit 59 to 73 days after flower development is suitable for extracting zygote, nucellus, and endosperm tissues for plant tissue culture breeding. The storage conditions can affect the calamondin fruit’s chemical composition and pericarp color. To maintain the chemical composition and color of the fruit, it is essential to store it under appropriate conditions. Good manufacturing practices, such as storing it in the dark and immersing it in benzyl adenine, a synthetic cytokinin, can slow down respiration, deterioration, and chlorophyll degradation. This method will help to slow down the color change of the calamondin fruit peel. Various post-harvest management techniques, such as soaking, pickling, drying, and cooking, are commonly used in Vietnam and the Philippines. Despite their small size, the fruits can be used in their entirety, from the peel and juice to the dregs, and are nutritious and can be processed into various products [6].

## 3. Chemical Composition and Bioactive Compound of Calamondin Fruit

Fresh calamondin fruit comprises two main components: juice, which accounts for 47.5 g, and byproducts from juicing (peel, seeds, and pulp), which account for 52.5 g. The byproducts are primarily composed of 80% liquid and 20% pulp. Each fruit provides 12 calories, with a low-fat content, 1.2 g of dietary fiber, 37 mg of potassium, 7.3 mg of vitamin C, 54.4 mg of vitamin A, and 8.4 mg of calcium [7]. Studies have also reported on the chemical composition of different parts of the calamondin fruit: the juice contains 0.10% total flavonoids, 6.74% total acid, 0.37% reducing sugar, and 1.68% vitamin E. The fruit pulp has flavonoids at 1.02%, pectin at 5.04%, limonin at 0.03%, and reducing sugar at 4.56%. The fruit peel contains 1.00% flavonoids, 7.14% pectin, 0.51% limonin, 5.98% reducing sugar, and 4.25% essential oils (volatile oils). The fruit seeds contain 0.46% limonin, reducing sugar, and 30.46% essential oils (volatile oils). The fruit dregs also contain 44.83 mg/kg of vitamin B2, 19.31 mg/kg of vitamin B3, 17.55 mg/kg of vitamin B6, and 15.72 mg/kg of vitamin E. The most common amino acids in the fruit residue, as reported by Zou et al. [8], are aspartic acid (4.42 g/kg), proline (3.65 g/kg), cysteine (2.10 g/kg), glutamic acid (1.81 g/kg), and isoleucine (1.00 g/kg) (Table 1). The fresh fruit has a unique flavor that combines the tastes of mandarin and lime with a slight hint of mandarin peel and tartness. Table 2 shows that the volatile intensity of the peel is three times higher than that of the juice.

Calamondin fruits contain ascorbic, dehydroascorbic, and citric acids as their primary acids. The peel and dregs of the fruit are high in dietary fiber, with a total of 84.25% dietary fiber, 48.49% insoluble fiber, and 35.76% soluble fiber [10]. Although not considered nutrients, the fibers from calamondin fruit play a significant role in the body by promoting regular bowel movements and helping to excrete food waste and toxins the body does not need. This can help to keep the intestines functioning normally and reduce the risk of colorectal cancer [1]. Soluble fiber can absorb a large amount of water and thicken food by increasing the viscosity. Once it reaches the small intestine during digestion, the viscous fiber thickens the intestinal wall, slowing the absorption of sugary foods and preventing sudden drops in blood sugar levels, known as acute hypoglycemia. Soluble fiber can also help reduce cholesterol levels and remove certain metal toxins from the body [11]. The fruit has medicinal properties, including its various organic acids. The fruit juice mixed with salt can alleviate coughing and phlegm, while the ripe fruit can be pickled, dried, or stored to treat sore throats. Raw fruit peels can also be used as a carminative [4,5,6]. Essential oil can be obtained from the peel of calamondin fruit, whether fresh, dried, or frozen. The fruit peel contains various important chemicals (limonene, α-pinene, β-pinene, linalool, geraniol, and citral) similar to those found in other citrus fruits of the Rutaceae family. The fruit peel also contains flavonoids like naringosi, hesperidin, diosmin, diosmetin, and hesperidin, which can protect the walls of blood vessels, reduce their permeability, and increase their elasticity, thus preventing blood vessel rupture and lowering blood pressure [1,12]. Essential oils extracted from the calamondin fruit peel are clear liquids with a distinct aroma. The essential oils of calamondin consist of 25 compounds, with D-Limonene being the most abundant, accounting for about 85% of the total (Table 3). The results of gas chromatograph–mass spectrometer (GC-MS) analysis showed a change in the composition of the calamondin peel oil, with limonene increasing from 90.4% to 92.87%. Santolina remained unchanged, α-terpineol was no longer present, and β-pinene increased from 1.01% to 1.03%, while other chemicals decreased. The improvement in oil content was due to refinement, resulting in a purer white color and distinct scent as unneeded components were removed and the limonene content increased. The essential oil has various benefits, such as preventing nail fungus, promoting restful sleep, treating headaches, stimulating appetite, deodorizing, reducing blood sugar levels, and relieving flatulence. It also can potentially prevent the development of liver, esophageal, colon, and skin cancers [12].

**Figure 2:**
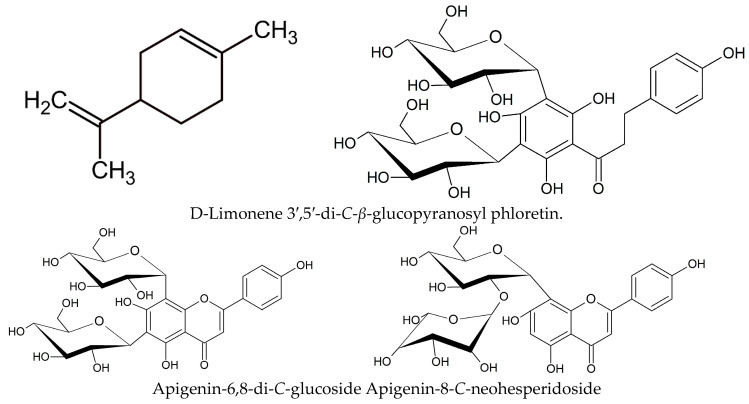
Chemical structures of D-Limonene; 3′,5′-di-*C*-*β*-glucopyranosyl phloretin; apigenin-6,8-di-*C*-glucoside; and apigenin-8-*C*-neohesperidoside in calamondin fruit peel.

D-Limonene (C_10_H_16_) is a hydrocarbon that appears as a clear liquid and is classified as a terpenoid (Figure 2). It belongs to the group of monoterpenes and can be found in various citrus plants, such as oranges and limes, on the surface of the fruit peel. D-Limonene is found naturally in a variety of citrus plants, such as limes, oranges, lemons, and kaffir limes, and is commonly used as a flavoring agent in the food industry, as well as in cosmetic products like shampoos, lotions, and soaps. The limonene can be extracted from the fruit peel using steam or organic solvent. D-Limonene can be extracted using various methods; the primary solvents used for extractions include hexane, methanol, and acetone, and the yield of D-limonene varies with the type of solvent used. D-Limonene has various beneficial biological properties, including anticarcinogenic activity, antioxidant activity, effects on apoptosis, and modulation of the immune system [12,13]. Phenolic compounds, such as flavonoids, possess nutraceutical properties that benefit health. Flavonoids are phenolic compounds containing an aromatic ring with two or more hydroxyl groups in their chemical structure. They are often found in combination with sugar moieties (glucose, rutinose, and neohesperidose) as glycoside compounds [13].

Currently, D-Limonene is used in various industries, which include the following:

1. Food processing, where it can be used as a mask for the bitter taste of alkaloids [14].
2. Flavorings for food products with a citrus or lime flavor, such as chewing gum, beverages, etc. [15].
3. Pharmaceutical applications, for use as a penetration enhancer for topical creams or lotions [16].
4. Perfumery in skin care products, such as lotions, body wash, and soap [17].
5. Natural insecticide flavorings [18].
6. Organic herbicide products [19].

**Figure 3:**
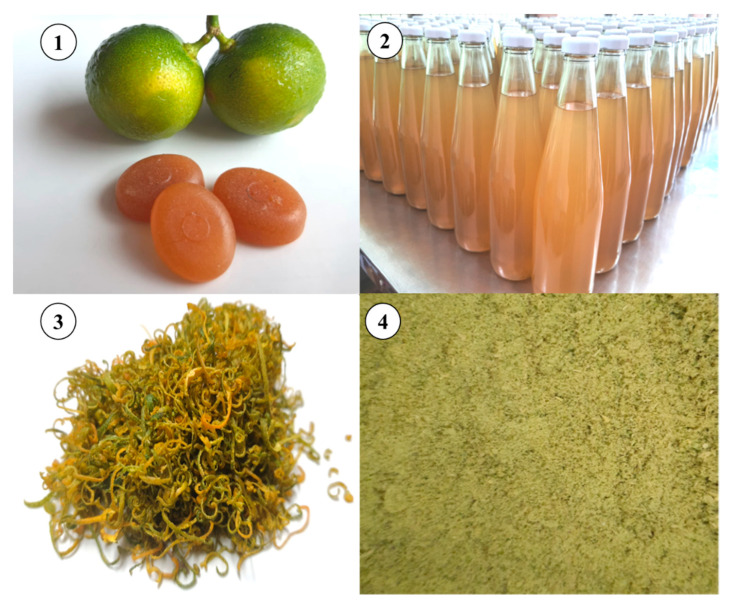
Calamondin and its processed products: (**1**) Calamondin candy, (**2**) Sterilized juice, (**3**) Dried peel, and (**4**) Residue powder.

Furthermore, the peel and juice sacs contain major flavonoids, such as 3′,5′-di-C-β-glucopyranosyl phloretin (DGPP), apigenin-6,8-di-C-glucoside (vicenin-2), and apigenin-8-C-neohesperidoside (Figure 3). Calamondin juice is widely used commercially, producing excess waste, such as seeds, pulp, and peel. These byproducts can be beneficial and help reduce environmental pollution when used as herbal medicine. The juice, peel, pulp, and seeds of calamondin have been found to exhibit various biological and pharmacological properties, including antioxidant activity and antimicrobial, anti-inflammatory, anti-hyperglycemic, anti-diabetic, anti-angiogenic, and anti-tumor properties, particularly in the peel [20,21,22,23,24,25,26,27,28,29,30,31] (as shown in Table 4).

## 4. Utilization of Calamondin Fruit

The calamondin fruit is used to produce a broad range of processed and non-food items (as illustrated in Figure 3). Calamondin fruit is used as a substitute for lime in various forms, such as freshly squeezed juice, mixed with other fruit juice, syrup, powder, jam, tea, and candy, and is used as a condiment in many savory and sweet dishes. The fibers extracted from the fruit can also partially replace wheat flour in the production of steamed bread. Incorporating 3–6% of dietary fiber powder from the fruit into steamed bread production results in more complex bread; however, it may also decrease the bread’s cohesiveness, specific volume, and elasticity. This proportion of fiber powder is considered an appropriate amount and results in bread products that are high in fiber, which is beneficial for health, as well as phytochemicals and antioxidant capacity [32]. In addition to being used for human consumption, the calamondin fruit is also utilized in cosmetics and skin care products. It is used in forms such as scrubs, massages, applications, rubs, masks, and various other forms for beauty enhancement. The properties of these cosmetics cannot cure, treat, relieve, or have a therapeutic effect on the skin, but they can be used to conceal various dark spots on the skin. Calamondin fruit is also used in cosmeceuticals, skin care products that have the same properties as cosmetics, but can also treat various skin disorders, such as acne, wrinkles, dark spots, and freckles. It also has a healing effect on the skin, similar to medicine [14,21,33].

The fruit is rich in vitamin C, polyphenols, and trace elements. Polyphenols, a type of phenolic compound found in plants, have the potential to scavenge free radicals due to their stable structure after binding to free radicals. The calamondin fruit peel is also rich in tangerine and sinensetin. The fruit extract possesses properties as an antioxidant and skin-whitening agent, making it suitable for use in cosmetic and cosmeceutical products, which enhances the effectiveness of the product [33]. Furthermore, calamondin fruit can also be used to eliminate dandruff, alleviate itchy scalps, promote hair growth, prevent respiratory diseases, stimulate blood circulation, aid in normal digestive function, alleviate symptoms of insect bites, relieve cough, expel phlegm, treat acne, and reduce wrinkles [34].

## 5. Examples of Calamondin Fruit Processing

### 5.1. Juice and Juice Concentrate

Calamondin juice has a total soluble solid content of 9.0°Brix, pH of 2.40–3.00, and citric acid of 4.50–5.80% [35]. The best juice is made from mature and sound fruit harvested optimally. The quality of calamondin juice depends on the quality of the fruit. To ensure high quality, the fruit must be fully mature, with the suitable sugar, acid, color, flavor, and firmness levels. Proper handling during harvest, transportation, and storage is crucial for preserving freshness and quality. To maintain the fruit quality and prevent decay, the temperature, humidity, and cleanliness must be regulated during storage to prevent damage from pests, bacteria, molds, yeasts, etc. [36]. Juice extraction can be done with machinery or by hand. For large-scale juice production, standard methods use calamondin fruit juice extractors. For small-scale juice production, pressing, centrifuging, or reaming extractors can be used. Home, retail, and manufacturing locations can access various small-scale juice extractors [35]. Calamondin juice is pasteurized to eliminate harmful and spoilage microorganisms since its acidic nature (pH 4.6) only needs to kill microorganisms in their vegetative state [36]. Research has been done on calamondin-based products, such as concentrated non-alcoholic syrups for beverages, made from calamondin juice, water, and sugar or sugar substitutes and may include added food coloring and flavoring [37]. A study examined the effect of storage time on calamondin fruit squash with 43°Brix and 60°Brix sweetness levels stored at room temperature. The study found that the storage time impacted the squash’s L* and b* values, and more sediment was observed in the 60°Brix sweetness level compared to the 43°Brix level. However, the storage time did not affect the total soluble solids and pH levels of the calamondin fruit squash. The titratable acidity and ascorbic acid levels in calamondin squash decreased as the storage time increased from 2 to 6 weeks. The total microorganisms, including yeast and molds, reached standard levels after ten weeks of storage [10]. A mocktail drink was made using calamondin juice and camote leaf extract, and its physiochemical and sensory qualities were evaluated. The mocktail was marketable, with the optimal composition consisting of 200 g refined sugar, 175 mL calamondin juice extract, and 1200 mL camote leaf extract. The mocktail met taste preferences when made as instructed, with good overall quality [38].

### 5.2. Pectin

Pectin is a polysaccharide found in the peel and pulp of calamondin fruits, and it functions as a natural cement to help the cell wall hold together. It comprises D-galacturonic acid, methyl lacturonate, and other sugars like rhamnose, galactose, and arabinose. Pectin makes up about 65% of the weight of the peel and pulp and plays a crucial role in maintaining the structural integrity of the fruit [39]. Extracting pectin from the skin of calamondin fruit involves several steps. First, the fruit’s peel, a byproduct of oil extraction, is dried and crushed. The resulting powder is then adjusted to a pH of 1 and treated with citric acid. The mixture is heated in a temperature-controlled bath at 80 °C for 10 min, after which it is filtered to separate the dregs. The filtered solution is then precipitated with ethanol and centrifuged at 4000 rpm for 15 min to obtain jelly pectin. The jelly pectin is then dried at 50 °C for 10 h to obtain pectin from the calamondin fruit peels. The highest yield of pectin from calamondin using ethanol as a solvent was 45.7%, indicating that calamondin pectin has the potential to replace halal gelatin in the food, cosmetics, and pharmaceutical industries [40]. Pectin from citrus fruit peels has various uses in the food industry, including gelling in jams/jellies with sugar and acid [41], thickening [42], stabilizing food products to prevent sedimentation [43], acting as an emulsifier [44], and serving as a prebiotic food for beneficial probiotics [45].

### 5.3. Tea

Calamondin fruit is commonly used as a raw material in producing various fruit teas and desserts, including calamondin tea [4]. The juice from the fruit is often used in this context due to its low sugar content, pungent aroma, and high levels of ascorbic acid, dehydroascorbic acid, and citric acid [46]. Calamondin fruit tea is made by steeping slices or peels of the fruit in boiling water. This type of tea is popular in Taiwan, particularly during the winter, as it has a warm, comforting aroma and a unique flavor. Additionally, the essential oils found in the fruit can provide a warming sensation when consumed [47]. The whole fruit contains monoterpene alcohols, such as linalool, terpinen-4-ol, and a lot of -terpineol, which can affect the flavor of calamondin fruit teas. The essential oil extracted from calamondin fruit peel has higher sesquiterpenes, such as germacrene D, than those found in whole calamondin fruit oils [48]. A study compared the methods of extracting essential oils from the whole calamondin fruit and its peel. It was found that using heat treatment in the extraction process increased the yield of essential oils from both the fruit and peel. The essential oils from the whole fruit contain monoterpene alcohols, such as linalool and terpinen-4-ol, which contribute to the unique flavor of calamondin fruit teas. The essential oils extracted from the peel have a higher concentration of sesquiterpenes, such as germacrene D, than the whole fruit [49].

### 5.4. Calamondin Fruit Powder

**Figure 4:**
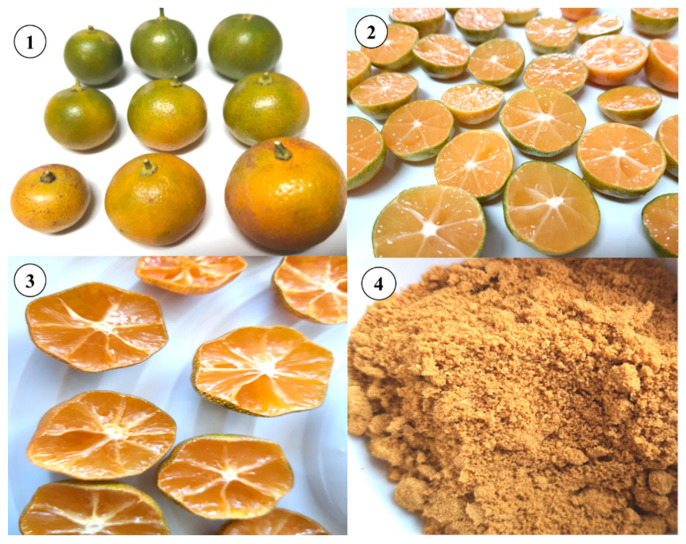
Calamondin powder: (**1**) grading, (**2**) cutting and removing seeds, (**3**) after dehydration, and (**4**) powder after using a grinder.

Calamondin fruit powder, with its high citric acid content and unique sour taste, is commonly used as a flavoring ingredient in a wide range of food and beverage products, including beverages, teas, cocktails, marinated meats, seafood, dairy products, and baked goods [35]. Due to its high antioxidant content, it can also be added to meat and fish products to prevent fat oxidation and extend shelf life [50]. The production of calamondin fruit powder involves several steps (Figure 4): (1) selecting fresh and fully ripe calamondin fruits to ensure good quality powder, (2) sorting and cleaning the fruits to remove any defects or impurities, (3) disinfecting the surface of the fruits to reduce the presence of pathogens, (4) cutting the fruits in half to facilitate water removal and seed removal, (5) drying the fruits in an oven at a low temperature, (6) grinding the dried fruits into a fine powder, (7) packaging the powder in sealed containers to prevent moisture absorption, (8) storing the powder in a cool and dry place, such as a refrigerator, for up to 6 months. This process can produce high-quality calamondin fruit powder with high antioxidant content, which can be used as a flavoring ingredient in food and beverage products and prevent fat oxidation in meat and fish products [40,51]. The leftover fruits from juicing can be dried and ground into powder, which can be used as an ingredient in body scrubs in the spa industry and other cosmetic products [52]. Several drying techniques are used to produce calamondin fruit powder. A study was conducted to determine the optimal conditions for producing the powder using spray drying and to examine its chemical and physical properties. The experiment involved preparing calamondin juice; blending it with maltodextrin at ratios of 10%, 20%, and 30%; and spray drying it at an inlet temperature of 170 °C and a feed rate of 20 RPM. The results showed that maltodextrin at a 10% concentration produced a yellow powder with a distinct calamondin odor and high vitamin C content [53]. The production of a ready-to-drink fruit beverage using calamondin juice and fluidized bed drying was studied. The process involved converting calamondin juice into a powder or agglomerate, adding citric acid, flavor, and color to enhance the flavor, acid–sugar balance, and appearance. The optimal process conditions were 45 °C process temperature, 20 m^3^/h airflow rate (1.5 m/s air velocity), 3 bars atomization pressure, and 6 g/min pump flow rate. The resulting fruit moisture content was 83.8%, and juice recovery was 35.8%. The analysis showed a total titratable acidity of 5.8%, pH of 2.4, total soluble solid of 8.5, and viscosity of 5.1 cP. The bulk density of the agglomerate was 0.6 g/mL, with a suitable particle size distribution. Compared to diluted calamondin juice, the calamondin drink performed better in a sensory evaluation [54].

### 5.5. Candy Products

Candy refers to sweet, flavored confections meant to be sucked or chewed. It typically contains sugar as the primary ingredient and may include additional flavorings and ingredients. The physical characteristics of candy can be classified into three types: hard candy, chewy candy, and soft candy [55,56,57]. An example of using calamondin juice to produce hard candy is shown in Figure 4. The process for making calamondin hard candy is as follows: (1) mix glucose syrup and sucrose with calamondin juice as a solvent and simmer, (2) stir until all components are homogenous, (3) increase the temperature to 120–130 °C to raise the sugar concentration, (4) quickly cool the solution down to 70–80 °C, (5) pour the mixture into molds and cool it to room temperature, and (6) package and wrap the candy immediately. A study on the optimal ratio of sucrose to glucose in the development of calamondin fruit candy found that the best ratio was 60% sucrose to 40% glucose. The calamondin fruit candy produced has a bright-yellow color, a citrus aroma and flavor, and a hard texture. Chemical analysis of the candy revealed that it has a moisture content of 1.80%, ash content of 0.15%, reducing sugar content of 68.03%, and total acid content of 2.28% [7]. The high temperatures in the production process can destroy the natural flavor, vitamin C, and other essential compounds in calamondin [58]. As a result, synthetic versions of these compounds are added to commercial candy production [59]. Additionally, low-calorie sweeteners or sugar substitutes, such as sorbitol and mannitol, may replace sugar in sugar-free candy formulas [60].

### 5.6. Essential Oils Extraction

**Figure 5:**
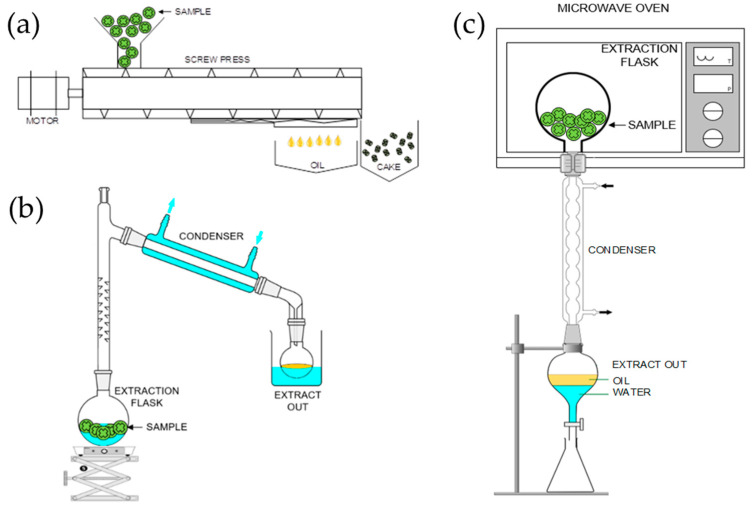
Schematic diagram: (**a**) screw press, (**b**) hydrodistillation, and (**c**) microwave-assisted distillation.

The essential oil derived from citrus fruits is an up-and-coming downstream product with significant potential for growth in the future. The increasing complexity and diversity of human demands, such as for use in the food additive, fragrance, cosmetics, and pharmaceutical industries, will likely drive demand for essential oils [61]. The limited size and thin skin of calamondin fruits may restrict the number of essential oils extracted from them [62]. However, increasing plant production can help maintain the high volume of raw materials necessary to produce essential oil from the peel of calamondin [63]. There are two main ways to increase plant production to meet consumer demand for essential oil: intensifying agricultural inputs and increasing the land used for cultivation. It should be noted that the rind of a mature (medium-ripe and fully ripe) calamondin fruit yields less essential oil than the peel of unripe fruit [61,62,63]. The amount of essential oil that can be extracted from ripe peels is typically lower than that from unripe peels because they are softer, and the biochemical content of the peel has changed [12]. Additionally, the origin of the plant and the method used for extraction can also affect the yield and composition of the essential oil [34,64,65]. Cold pressing or screw presses are traditional methods for extracting essential oils from citrus peels. In some countries, distillation is also commonly used due to its higher yield (0.21% compared to 0.05% for cold pressing) [66]. Modern methods of essential oil extraction include ultrasound-assisted extraction (UAE), microwave-assisted extraction (MAE), supercritical fluid extraction (SFE), and accelerated solvent extraction (ASE) [67,68,69]. These methods have been developed and improved over time and are widely accepted by the industry. The extraction of essential oils from calamondin fruit peel can be done using methods that have the potential to improve the efficiency, safety, energy savings, and sustainability. These methods include the following (Figure 5):

(1) A screw press uses mechanical force to extract essential oils from plants by breaking down the bulbous cells that store the oils [63]. Extracting essential oils from calamondin fruit peel using a screw press involves reducing the size of the peel with a rough chopping machine and then compressing it with a cold oil screw press. The output of the screw press is divided into two parts: a solid, or dregs, and a liquid containing essential oils. The essential oil is then separated from the other liquids [65]. Using this method, it is possible to extract one kilogram of lime essential oil from the peel by crushing the lime peel and oil glands and allowing the liquid to flow out of the bottom of the machine [70].
(2) Distillation with water, also known as hydrodistillation, uses the principle of pressure extraction from hot water to extract essential oils from plants by breaking down the bulbous cells that store the oils. The process of extracting essential oils from calamondin fruit peel using hydrodistillation involves reducing the size of the peel with a crushing machine, placing it in a round-bottom flask, filling it with water, and boiling it [63,65]. The hot steam carries the essential oil out and condenses it into a liquid. The hydrodistillation process involves heating the round-bottom flask containing the crushed calamondin fruit peel and water until it boils and becomes steam-containing volatile substances. The steam is then cooled to condense it into a liquid, from which the essential oils are separated. Hydrodistillation is a traditional method used in laboratory-scale extraction of plant essential oils. The hydrodistillation process was carried out by adding 50 g of crushed calamondin fruit peel to a flask, along with 250 milliliters of distilled water (at a ratio of 1 g of solids to 5 milliliters of water). The flask was then heated to boiling under atmospheric pressure to initiate the extraction. During the distillation process, volatile aroma compounds and water mix to form azeotropic mixtures and condense. The mixture is then stratified based on the difference in density. Due to incompatibility, the essential oil fractions are collected and separated from the water after 2 h of distillation [71].
(3) Microwave-assisted distillation uses the principle of electromagnetic extraction and the polar properties of molecules within the sample to extract essential oils. The movement caused by microwaves generates friction and heat, affecting plants’ cellular tissues [64]. To extract essential oils from calamondin fruit peel using this method, the peel is first reduced in size by crushing it. After crushing, the calamondin fruit peel is placed in a round-bottom flask and then in a microwave. The microwaves cause friction between the polar molecules, which generates heat and causes the liquid to boil into steam containing essential oils [68]. The steam is then cooled until it condenses back into a liquid, from which the essential oils are separated. Finally, the essential oils are separated from other liquids using a combination of microwave-assisted distillation. This process was carried out in a laboratory microwave oven at atmospheric pressure. A constant power was set in the microwave, and 500 g of fresh calamondin fruit peels were heated at 500 watts for 15 min. [66]. After that, the essential oil fraction was dried with Na_2_SO_4_, gathered in a sealed vial, and kept chilled at 4 °C.

Table 5 illustrates the results of the extraction of essential oils from calamondin fruit peel using different extraction methods and conditions. The data show that when compared to steam distillation alone, using a combination of hot water treatment followed by steam distillation led to an increase in the yield of essential oils for calamondin peels. However, it should be noted that hot water treatment followed by steam distillation may cause damage to the tissues in the calamondin peel or the flesh, releasing volatile compounds.

## 6. Possibility of Adding Commercial Fruit Value

**Figure 6:**
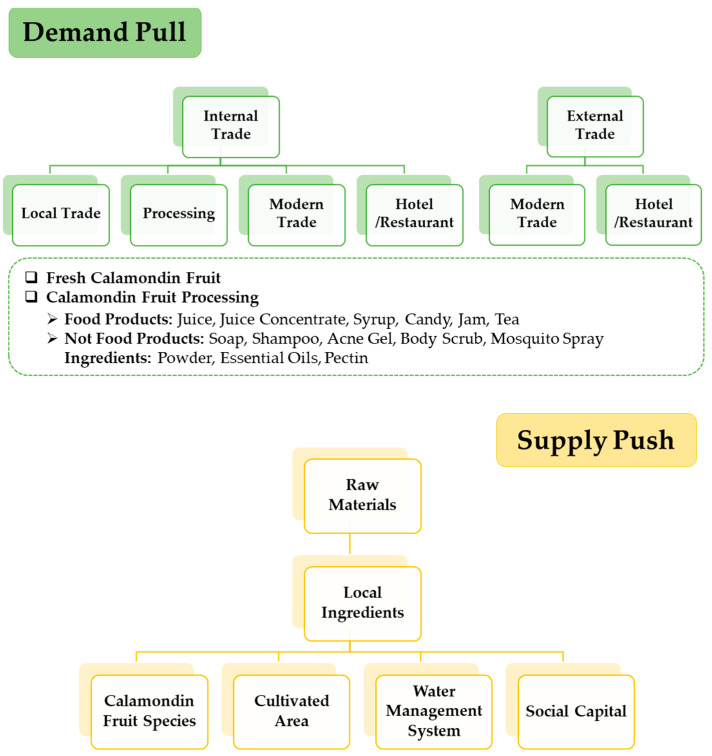
Demand and supply of calamondin fruit for commercial scale.

As mentioned earlier, calamondin is a citrus plant native to China and has been widely introduced to various countries in East and Southeast Asia, the Pacific Islands, the USA (Hawaii and Florida), and Central America [69]. In the past, calamondin was not considered a commercial citrus crop; it was primarily grown as an ornamental plant or a rootstock for citrus grafting [70,71]. However, it has become a commercial crop in recent years due to its fruits’ pleasant aroma, flavor, and nutritional value [72,73]. As a result, the global export value of calamondin has increased from USD 111.6 million in 2014 to USD 114.4 million in 2021 [74]. Calamondin fruit is a versatile and nutritious plant that can meet the needs of health-conscious consumers. It also can create added value as it can be used as an excellent substitute for limes when they are in short supply or expensive. Calamondin fruit has a higher juice yield than lime, with an 80% juice content compared to 40% for lime. In the Thai market, for example, the regular selling price of calamondin is USD 0.6 per kg, but during periods of high lime prices, such as November–January, the price of calamondin may increase to as much as USD 1.8 per kg. The domestic market demands up to 1000 kg of calamondin fruit per day, and it has a high demand in the international market, such as Singapore, which imports the fruit from Malaysia and the Philippines [75]. Calamondin is a commercially viable crop that is easy to grow, care for, and maintain. It produces fruit all year round and is rarely affected by disease or insect disturbances. This makes it a popular side crop, often planted around rubber trees and other local crops. Due to the increasing demand for fresh and processed products domestically and internationally, cultivation areas for calamondin have been expanding. It is commonly grown in mixed orchards alongside Thailand’s rubber, durian, and mangosteen trees. It is a versatile and nutritious plant that can be grown commercially and is relatively easy to care for. The cost of cultivation is determined by the planting distance, with the lowest price averaging USD 371 per 1600 square meter. Additionally, it has byproducts from providing water, fertilizer, and pest control from the main crop or natural release, making it popular to grow as a side crop with other local crops [76]. When analyzing the market for calamondin fruit, it is essential to consider both the pull–demand side and the supply–push side (Figure 6). As the demand for fresh and processed products increases at local, provincial, national, and international levels, so does the cultivation of calamondin. This corresponds to the context of Thai society, where fruit is used for both consumption and non-consumer purposes, such as cosmetics. The cultivation of calamondin fruit offers a wide range of benefits, both economically and socially. The growing demand for fresh and processed products, locally and internationally, has increased cultivation areas [73]. The low cost of cultivation and the ability to grow it as a side crop with other plants makes it an ideal crop for smallholder farmers with low-occupancy areas. Furthermore, the production and processing of calamondin products, such as juice, concentrate, and jam, can create jobs and income for farmers and communities, providing stability and prosperity. The Bio-Circular-Green (BCG) economic model is a model of the economy that emphasizes sustainable growth. Since the start of the privatization of agricultural raw materials cultivation, it has added value by paying close attention to every step of the agricultural raw materials production process and using knowledge, technology, and innovation to achieve the efficiency and effectiveness of the business cycle. The BCG model meets at least five Sustainable Development Goals (SDGs) of the United Nations: sustainable production, consumption, tackling climate change, conservation of diversity, and cooperation for sustainable development [77,78]. Additionally, the cultivation of calamondin aligns with the bio-circular-green (BCG) economic policy framework, as it utilizes the fruit’s juice, pulp, and peel to add value and reduce agricultural waste. This helps sustain the environment and supports the local economy by creating jobs and income for farmers and communities.

## 7. Conclusions

Calamondin fruits are an incredibly versatile and beneficial citrus fruit that offer a range of nutritional and economic advantages. These small fruits can be processed into various economically valuable products, such as juices, jams, and flavorings, and are a popular substitute for limes in many cuisines. Furthermore, calamondin cultivation is suitable for both small-holder farmers and larger-sized farmers, making it a profitable option for agriculture. The juice, pulp, seeds, peel, and fruit residue of calamondin fruits contain a rich chemical composition and bioactive compounds, offering a range of potential applications in several industries. These compounds have been shown to possess antioxidant, anti-inflammatory, and antimicrobial properties, among other health benefits, making them a valuable resource for food and beverage production, pharmaceuticals, and cosmetics. Additionally, utilizing different parts of the fruit, such as the peel and seeds, can minimize agricultural waste and add value, supporting the Bio-Circular-Green (BCG) economic model. By promoting a circular economy, we can reduce waste and environmental impacts, while also creating economic opportunities for communities. Overall, the cultivation and processing of calamondin fruits offer a sustainable approach to economic development, social progress, and environmental preservation. Utilizing calamondin fruits in various industries and promoting a circular economy can lead to the creation of economic and social benefits for communities, while simultaneously preserving the environment.

## Figures and Tables

**Table 1:** Chemical composition of various parts of calamondin fruit [8].

Compound	Content				
	Juice	Seed	Peel	Pulp	Fruit Residue
Total flavonoids	0.11 (%)	-	1.00 (%)	1.02 (%)	-
Pectin	-	-	7.14 (%)	5.04 (%)	-
Limonin	-	0.46 (%)	0.51 (%)	0.03 (%)	-
Reducing sugars	0.37 (%)	1.39 (%)	5.98 (%)	4.56 (%)	-
Volatile oils	-	-	4.25 (%)	-	-
Aspartic acid	-	-	-	-	4.42 (g/kg)
Proline	-	-	-	-	3.65 (g/kg)
Cystine	-	-	-	-	2.10 (g/kg)
Glutamic acid	-	-	-	-	1.81 (g/kg)
Isoleucine	-	-	-	-	1.00 (g/kg)
Lysine	-	-	-	-	0.91 (g/kg)
Serine	-	-	-	-	0.78 (g/kg)
Leucine	-	-	-	-	0.76(g/kg)
Arginine	-	-	-	-	0.72 (g/kg)
Alanine	-	-	-	-	0.67 (g/kg)
Valine	-	-	-	-	0.67 (g/kg)
Glycine	-	-	-	-	0.59 (g/kg)
Methionine	-	-	-	-	0.59 (g/kg)
Phenylalanine	-	-	-	-	0.57 (g/kg)
Tyrosine	-	-	-	-	0.50 (g/kg)
Histidine	-	-	-	-	0.20 (g/kg)
Threonine	-	-	-	-	0.16 (g/kg)
Total acid	6.74 (%)	-	-	-	-
Volatile oils	-	30.46 (%)	-	-	-
Vitamin B_2_	-	-	-	-	44.48 (mg/kg)
Vitamin B_3_	-	-	-	-	19.31 (mg/kg)
Vitamin B_6_	-	-	-	-	17.55 (mg/kg)
Vitamin E	1.68 (mg/kg)	-	-	-	15.72 (mg/kg)

Note: (-) indicates unavailability of the data.

**Table 2:** Flavor profiles of fresh calamondin peel and juice [9].

Total Volatile Intensity	Fresh Calamondin Peel (%)	Fresh Calamondin Juice (%)
Limonene	10.53–27.85	14.51–14.59
(*Z*)-3-hexenol	4.85–12.51	0.17–1.36
Linalool	9.40–10.29	-
1-octanol	2.55–2.84	-
α-terpineol	4.00–7.80	2.29–3.76
Isopiperitenone	1.91	0.28–0.76
Geraniol	0.79–1.06	-
8-hydroxylinalool	1.20–2.12	-
(*Z*)-8-hydroxylinalool	-	0.45–3.58
(*E*)-*ρ*-mentha-2,8-dien-1-ol	0.39–1.61	-
Hexadecanoic acid	0.81–1.31	3.19–10.88
4-hydroxy-benzeneethanol	-	0.09–7.98
Cryptomeridiol	0.26–069	4.95–5.76
Stearic acid	0.19–0.43	3.38–3.82
α-cadinol	-	1.23–3.16
Limonen-1,2-diol	-	0.41–2.85
Linoleic acid	-	1.36–2.73

Note: (-) indicates unavailability of the data.

**Table 3:** Ingredients of crude and refined calamondin peel oil [12].

Essential Oils	Crude (%)	Refined (%)
D-Limonene	90.40	92.87
β-Pinene	3.36	3.36
α-Pinene	1.01	1.03
β-copaene	0.91	0.90
1,6,10-Dodecatrien-3-ol, 3,7,11-trimethyl-, [S-(Z)]-	0.57	-
Santolina triene	0.54	-
Carvone	0.53	0.49
2,6-Octadien-1-ol, 3,7-dimethyl-, acetate	0.49	0.16
Octadecane, 6-methyl-	0.41	0.27
α-Terpineol	0.38	-

Note: (-) indicates unavailability of the data.

**Table 4:** Bioactive compounds of calamondin juice, peel, pulp, and seeds and their bioactivities.

Bioactive Activities	Calamondin Parts	Component	References
Antioxidant	Peel	Naringin and Hesperidin	[20]
Antioxidant	Peel, Pulp	Phenolics and Flavonoids	[21]
Anti-hepatitis B virus	Peel	Nobiletin, tangeretin and 5-hydroxy-6,7,8,3′,4′-pentamethoxyflavone	[22]
Antimicrobial	Peel	Flavonoids	[1]
Antimicrobial	Peel	Essential oil	[23]
Antimicrobial	Peel	Tannins	[24]
Anti-inflammatory	Peel	Tannins	[25]
Tyrosinase inhibitory	Peel	3′,5′-di-*C*-*β*-glucopyranosyl phloretin, Hesperidin and Neohesperidin	[26]
Anti-hyperglycemia	Peel	Flavonoids	[27]
Anti-diabetic	Peel	Phenolics	[28]
Anti-angiogenic	Peel	Phenolics	[29]
Antimicrobial	Peel, Seeds, Pulp	Flavonoids	[30]
Antioxidant	Juice	Phenolics	[31]

**Table 5:** Comparison of essential oils extracted from calamondin fruits by various methods.

Extraction Method	Conditions	Essential Oil Yield	Reference
Steam-enticing distillation	Raw material/solvent ratio (*w*/*v*) of 100/800, extraction time of 70 min.	2.3913 g/kg	[12]
Cold pressing	Sugarcane squeezer used to squeeze 1 kg of peel and centrifugation at 6000× *g* at 4 °C for 40 min.	0.81 ± 0.25 g/kg	[49]
Steam distillation (SD)	Peels (150 g) homogenized for 2 min with 600 mL deionized water and placed in a 5 L round-bottom flask. Homogenate steam distilled for 2 h.	7.11 ± 0.08 g/kg	[49]
Hot water treatment followed by steam distillation	Peels (150 g) heated in 90 ± 3 °C water for 15 min, homogenized for 2 min with 600 mL deionized water in a 5 L round-bottom flask, followed by SD for 2 h.	9.13 ± 0.12 g/kg	[49]
Microwave-assisted hydrodistillation (MAHD)	Extraction with MAHD in optimal conditions for 45 min, 300 W capacity, and 1:3 shells/water ratio.	2%	[68]

## Data Availability

Not applicable.
